# Sex differences in subjective cognitive impairment and clinical correlates in Chinese patients with subthreshold depression

**DOI:** 10.1186/s13293-023-00488-w

**Published:** 2023-02-13

**Authors:** Qinyu lv, Xin Li, Yao Zhang, Daofeng Lu, Jiajing Lu, Qingfang Xie, Hong Li, Yiming Wu, Chongze Wang, Zhenghui Yi

**Affiliations:** 1 Shanghai Mental Health Center, Shanghai Jiao Tong University School of Medicine, 600 South Wanping Road, Shanghai, 200030 China; 2 Institute of Mental Health, Fudan University, Shanghai, 200040 China; 3 Department of Psychiatry, Huashan Hospital, School of Medicine, Fudan University, No. 12 Wulumuqi Road (Middle), Shanghai, 200040 People’s Republic of China; 4 Shanghai Baoshan Mental Health Center, Shanghai, 201900 China; 5 Shanghai Yangpu Mental Health Center, Shanghai, 200093 China

**Keywords:** Subthreshold depression, Depressive symptoms, Cognitive impairment, Alexithymia, Sex differences

## Abstract

**Objective:**

Subthreshold depression (SD) is a global mental health problem given its high prevalence, comorbidity, functional impairment, and its association with increased service utilization. However, currently little is known about sex differences of SD in cognitive impairment with clinical correlates. This study aims to explore sex differences in subjective cognitive impairment and clinically associated risk factors in Chinese patients with subthreshold depression (SD).

**Methods:**

A total of 126 patients with SD, 40 males and 86 females, aged 18–45 years, were included in this cross-sectional observational study. Their general information, psychological assessments, and psychiatric symptom assessments were collected online. The Patient Health Questionnaire depression-9 (PHQ-9), Generalized Anxiety Disorder-7 (GAD-7), Perceived Deficits Questionnaire-Depression (PDQ-D), and Toronto Alexithymia Scale (TAS-20) with 3 subdomains were used. The obtained scores were analyzed with partial correlation and multiple linear regression analysis models.

**Results:**

Our results showed that females had significantly higher PDQ-D-20 total score than males. However, the differences in TAS-20 and subdomain score according to sex were not significant. Notably, TAS-20 and DDF (difficulty describing feelings) subdomain contributed to cognitive impairment in males, whereas both PHQ-9 total score and TAS-20 or DDF subdomain contributed to cognitive impairment in females.

**Conclusion:**

These findings revealed significant sex differences in cognitive impairment and clinical correlates in SD, which should be further followed-up in the future.

**Supplementary Information:**

The online version contains supplementary material available at 10.1186/s13293-023-00488-w.

## Highlights

This study is the first to explore sex differences in subjective cognitive impairment and clinical associated risk factors in Chinese patients with subthreshold depression.

Notably, TAS-20 and DDF (difficulty describing feelings) subdomain contributed to cognitive impairment in males, whereas both PHQ-9 total score and TAS-20 or DDF subdomain contributed to cognitive impairment in females.

**Supplementary Information:**

The online version contains supplementary material available at 10.1186/s13293-023-00488-w.

## Introduction

In recent decades, subthreshold depression (SD) has gained considerable attention due to its high prevalence in the population. Thus far, different terms have been used to describe this condition, such as subclinical, subsyndromal, or minor depression; however, SD refers to individuals with clinically relevant depressive symptoms that fall short of the criteria for a major depressive disorder (MDD) [1]. MDD is diagnosed when there are at least 5 out of 9 criteria symptoms for depression lasting minimally for 2 weeks, 1 of which is depressed mood or anhedonia. In comparison, SD is diagnosed when 2–4 criteria symptoms for depression and 1 core symptom, such as depressed mood or anhedonia are present for 2 weeks or longer [1, 2]. Even though SD is characterized by less severe symptoms than MDD, its health service utilization is greater than MDD on a population basis. Zhang et al. [3] conducted a meta-analysis of data from 113 studies covering 1,129,969 individuals, finding a summary prevalence of 11.02%. The prevalence in the youth group (aged < 18), the adult group (aged 18–60), and the elderly group (aged > 60) was 14.17%, 8.92%, and 12.95%, respectively. Moreover, individuals with SD are more vulnerable to developing MDD [3, 4]. Previous studies have shown that individuals with SD report moderate functional impairment, have poorer quality of life [5, 6], and tend to cope with enormous economic costs because of disability days [7].

Cognitive impairment is one of the major characteristics of patients suffering from depression. Accumulating evidence shows that cognitive symptoms in depressed patients lower their physical and mental efficiency. In addition, cognitive symptoms are commonly reported in both the acute phase and the remission of depressive symptoms. A large cross-sectional study conducted in six Asian countries showed that approximately 67.4% of medication-free outpatients with depression suffered from subjective memory deficits, and 73.2% suffered from subjective concentration deficits. It has been reported that subjective and objective cognition impairments further contribute to disability in patients with depression [8]. In recent decades, many studies have focused on the impact of cognitive symptoms such as slow thinking, lack of concentration, distractibility, memory problem, and decision-making difficulty on functioning [9]. For example, McIntyre et al. have reported that subjective cognitive dysfunction contributed more to poor workplace performance than a depressive symptom. However, far less attention has been paid to cognitive deficits in patients with SD. Hwang et al. [10] found that SD was associated with impaired resting-state functional connectivity of the cognitive control network, which is involved in cognitive processing (memory impairment, difficulties in decision making, and cognitive inflexibility) and cognitive biases (negative thoughts). Another study reported that implicit emotional neurocognitive processing was impaired in college students with SD.

Previous studies have found that women had a higher prevalence of SD than males [11, 12]. The meta-analysis showed that the estimates of SD in females (13.8%) were significantly higher than in males (9.68%) [3], which was similar to MDD [13]. Bennett et al. found gender differences in types of symptoms among depressed patients, with females being more likely to experience more depressed mood, appetite, and sleeping problems than males [14]. Another research found that girls exhibited more depressive mood and sleeping problems, whereas boys displayed higher levels of anhedonia, concentration problems, and psychomotor dysregulation. Also, girls performed worse than boys on variables such as social problem-solving and emotion regulation [15]. However, sex differences in cognitive deficits in patients with SD remained unclear.

Gaining a better understanding of sex differences in cognitive impairment in patients with SD is relevant, as it can affect treatment options and responses. In the present study, we aimed to explore sex differences in subjective cognitive impairment and clinically associated risk factors in Chinese patients with SD, which has not yet been assessed in patients with SD.

## Methods

### Subjects

This cross-sectional observational study was conducted at the psychological consultation clinic and outpatient service of Shanghai Mental Health Center, Shanghai Jiao Tong University School of Medicine, Shanghai city, China, between November 1, 2021, and January 31, 2022. This study was approved by the Ethics committee of the Shanghai Mental Health Center (2021-49).

Convenience sampling methods were employed, and a QR code was distributed to collect all the information. Online psychological assessments included general information, alexithymia assessments, subjective cognitive assessment, and psychiatric symptom assessments (depression, anxiety). General information related to sex, age, education level, career, marital status, and history of smoking or drinking were collected. Participants were informed of the purpose of this study before assessments. All participants provided electronic informed consent. All items were set as required questions. Submitting a visiting serial number indicated the completion of the questionnaire.

Inclusion criteria were the following: (1) age between 18 and 45 years; (2) SD referred to a depressive state where patients had certain depression symptoms without meeting the criteria for major depression according to the International Classification of Diseases, 10th edition (ICD-10).

Exclusion criteria were: (1) those who met the ICD-10 criteria for MDD, mild depressive disorder or dysthymia; (2) major medical, neurological diseases; (3) substance dependence/abuse; (4) pregnancy or lactation; (5) with major psychotic disorder (schizophrenia, bipolar disorder).

### Data collection and assessment

Depression symptoms were evaluated using the Patient Health Questionnaire depression (PHQ-9), which contained 9 items, each one ranging from 0 to 3. Depression Severity was ranked based on the total scores: normal (0–4), mild depression (5–9), moderate depression (10–14), moderate to severe depression (15–19), and severe depression (20 or greater). The Cronbach’s α coefficient of the Chinese version of the PHQ-9 and the test–retest reliability was reported to be 0.86 and 0.86, respectively. In the present study, Cronbach's α was 0.87, indicating a good internal consistency of these measurements.

The severity of anxiety was evaluated using the Generalized Anxiety Disorder-7 (GAD-7) scale, which contained 7 items, each one ranging from 0 to 3. Anxiety Severity was ranked based on the total scores, where 0–4 (no anxiety), 5–9 (mild anxiety), 10–14 (moderate anxiety), and 15–21 (severe anxiety). The Cronbach’s alpha coefficient of the Chinese version of the GAD-7 was 0.89, and the test–retest reliability was 0.85. In this study, Cronbach's α was 0.88, indicating a good internal consistency of these measurements.

Subjective cognitive deficits were evaluated using Perceived Deficits Questionnaire-Depression (PDQ-D), which included 20 items, and ranged from 0 (never in the past 7 days) to 4 (very often, more than once a day). The PDQ-D-20 evaluated four domains of cognitive function, including retrospective memory, attention/concentration, prospective memory, and planning/organization. Higher scores indicated a greater degree of cognition symptoms. The Chinese version of the PDQ-D-20 has been previously validated, revealing satisfactory reliability (Cronbach’s alpha coefficient = 0.948). In this study, Cronbach’s α was 0.9 which indicated a good internal consistency of these measurements.

In the present study, 20-item Toronto Alexithymia Scale (TAS-20) was used for assessing alexithymia. The TAS-20 consisted of three subscales for the subcomponents of alexithymia, including (1) difficulty identifying feelings and distinguishing them from bodily sensations of emotion (DIF), (2) difficulty describing feelings to others (DDF), and (3) externally oriented thinking (EOT). The TAS-20 included 20 items, where a score of each item ranged from 1 (totally disagree) to 5 (totally agree). Alexithymia severity was ranked based on the TAS-20 total scores, where ≤ 51 (no alexithymia), 52–60 (borderline alexithymia), and ≥ 61 (alexithymia). The TAS-20 has been validated and showed satisfactory reliability (Cronbach’s alpha coefficient ≥ 0.7). The Cronbach’s alpha coefficient of the Chinese version of the TAS-20 was 0.83, and the test–retest reliability was 0.87 [16].

### Statistical analysis

SPSS version 25.0 (IBM SPSS Statistics for Macintosh, Armonk, NY, USA) was used to perform all the statistical analyses. The Kolmogorov–Smirnov test was used to test the normality of the distribution. All continuous data were expressed as the mean ± standard deviation (SD), while independent sample *t*-tests and Mann–Whitney *U* test was used for group comparisons; Chi-square test or Fisher’s exact test were employed for categorical variables. In addition, the correlation of PDQ-D-20 total score and clinical variables was assessed with partial correlation coefficients. Bonferroni corrections were used for multiple comparison corrections. Next, multiple linear regression analyses were conducted to identify characteristics related to cognitive impairment in all participants and the male and female groups separately. We also conducted multivariable regression analyses, where the PDQ-D-20 score was taken as the dependent variable. The following independent variables were entered into the model with the enter selection procedure: sex, age, GAD-7, PHQ-9, and TAS-20 total score. According to previous studies, these factors may have an impact on cognition [9, 17–20]. As the smoking rate differed between the male and female groups, smoking was controlled as a covariable. Sex by PHQ-9 and TAS-20 score interaction was added to the equation model. The sex subgroup was included in the equation with age, smoking, GAD-7, PHQ-9, and total score of TAS-20 as independent variables. We also performed multiple regression analyses as an exploratory approach considering the TAS-20 subscale (DIF, DDF, EOT) along with the same covariates in relation to subjective cognitive impairment. When there was collinearity between independent variables, the stepwise regression method was used. All statistical tests were two-sided, and *p* values < 0.05 were considered statistically significant.

## Results

### Social-demographic and clinical characteristics

Among a total of 200 subjects who were initially enrolled in the study, 23 participants did not complete cognitive function assessment (declaring it as meaningless or not finishing all processes), 20 patients were excluded due to age mismatch, and 28 patients refused to participate in the research (having no time or not being able to cooperate), resulting in 129 subjects who completed the whole assessments. In addition, a box-plot was performed, and 3 cases of PDQ-D-20 outliers were found. Finally, 126 people, 40 males and 86 females, were included in this study. In our study, the PDQ-D-20 total score of the patients was 32.03 ± 14.97, and their average age was 26.31 ± 7.22 years.

As shown in Table 1, the mean age in the male and female groups was 26.20 ± 6.63 years vs. 26.36 ± 7.52 years, respectively. Fewer female (2.3%) reported smoking compared to male (22.5%) (*p* = 0.001). The mean PDQ-D-20 total score of patients with SD in this study was 32.03 ± 14.97, being significantly higher in females than males (34.21 ± 15.00 vs. 27.35 ± 13.95) (*F* = 5.962, *p* = 0.016). Except for smoking and PDQ-D-20 total score, there were no significant differences in demographic and clinical characteristics between male and female patients. Therefore, smoking was controlled in the following analysis that compared sex differences in subjective cognitive impairment.

**Table 1:** Social-demographic information and clinical characteristics of male and female patients with SD

Variables	Total	Male (*n* = 40)	Female (*n* = 86)	*t*/χ^2^/Fisher	*p* value
Marital status					
No married	87	28 (70.0%)	59 (68.6%)	–	0.728
Married	36	12 (30.0%)	24 (27.9%)		
Divorced/Widowed	3	–	3 (3.5%)		
Educational background					
High school or lower	23	8 (20.0%)	15 (17.4%)	4.307	0.116
College	75	19 (47.5%)	56 (65.1%)		
Graduate or above	28	13 (32.5%)	15 (17.4%)		
Career					
Worker	6	4 (10.0%)	2 (2.3%)	4.217	0.239
Staff	55	18 (45.0%)	37 (43.0%)		
Student	51	15 (37.5%)	36 (41.9%)		
Unemployed	14	3 (7.5%)	11 (12.8%)		
Smoking				–	**0.001**
No	115	31 (77.5%)	84 (97.7%)		
Yes	11	9 (22.5%)	2 (2.3%)		
Drinking				–	1.0
No	124	39 (97.5%)	85 (98.8%)		
Yes	2	1 (2.5%)	1 (1.2%)		
Age		26.20 ± 6.63	26.36 ± 7.52	0.116	0.908
GAD-7 score		8.03 ± 5.62	7.71 ± 3.91	0.321	0.749
PHQ-9 score		10.18 ± 4.03	9.99 ± 4.29	0.231	0.817
DIF		20.05 ± 5.50	21.20 ± 5.60	1.076	0.284
DDF		14.48 ± 2.72	15.00 ± 2.57	1.048	0.297
EOT		26.13 ± 2.81	26.59 ± 3.21	0.791	0.430
TAS-20 score		60.65 ± 7.62	62.79 ± 8.35	− 1.376	0.171
PDQ-D-20 score		27.35 ± 13.95	34.21 ± 15.00	− 2.442	**0.016**

*GAD-7* Generalized Anxiety Disorder-7, *PHQ-9* Patient Health Questionnaire depression-9, *TAS-20* 20-item Toronto Alexithymia Scale, *PDQ-D-20* 20-item Perceived Deficits Questionnaire-Depression, *DIF* Difficulty Identifying Feelings, *DDF* Difficulty Describing Feelings, *EOT* Externally Oriented Thinking

Bold values indicate statistical significance

### Association between subjective cognition and clinical variables

**Fig. 1:**
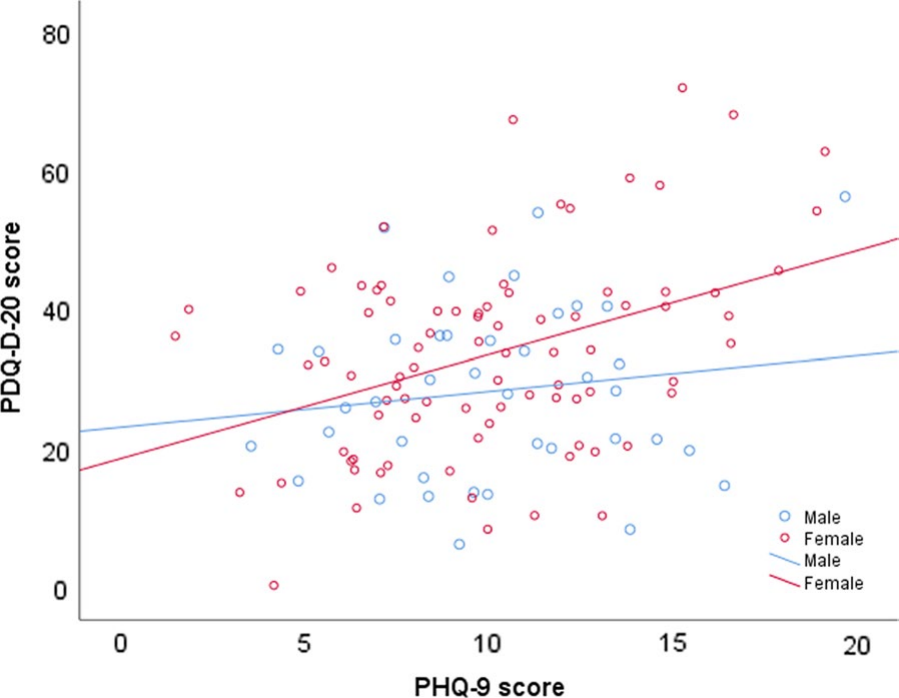
Relationship between 20-item Perceived Deficits Questionnaire-Depression (PDQ-D-20) score and Patient Health Questionnaire depression-9 (PHQ-9) score in males and females. Age, Generalized Anxiety Disorder-7 (GAD-7), 20-item Toronto Alexithymia Scale (TAS-20), and smoking were adjusted as covariates

For all patients, sex (*β* = 6.153, *p* = 0.025), TAS-20 score (*β* = 0.506, *p* = 0.001), and PHQ-9 score (*β* = 1.217, *p* < 0.001) were significantly associated with the multiple linear regression model (*R*^2^ = 0.260, adjusted *R*^2^ = 0.223, *p* < 0.001). The multivariable linear regression results are shown in Table 2. Furthermore, the sex*PHQ-9 and sex*TAS-20 score interaction terms were added in a multiple regression model with a stepwise selection procedure, revealing that sex*PHQ-9 (*β* = 0.632, *t* = 4.628, *p* < 0.001, 95% CI 0.362 to 0.902) and TAS-20 score (*β* = 0.506, *t* = 3.465, *p* = 0.001, 95% CI 0.217 to 0.796) were significantly associated with PDQ-D-20 score, and thus indicating that the relationship between PHQ-9 score and the subjective cognitive impairment differs between males and females. This interaction is depicted in Fig. 1.

**Table 2:** Multiple linear regression results of PDQ-D-20 total score for all participants

Independent variable	*β*	*SE*	*β’*	*t*	*p*	*β* 95%CI
(Constant)	− 17.231	12.212		− 1.411	0.161	− 41.413, 6.951
Age	− 0.113	0.172	− 0.054	− 0.654	0.515	− 0.453, 0.228
Sex	6.153	2.706	0.192	2.274	**0.025**	0.795, 11.510
Smoking	1.027	4.468	0.019	0.230	0.819	− 7.821, 9.875
GAD-7 score	− 0.240	0.298	− 0.072	− 0.805	0.423	− 0.831, 0.351
PHQ-9 score	1.217	0.321	0.341	3.796	**< 0.001**	0.582, 1.852
TAS-20 score	0.506	0.154	0.276	3.285	**0.001**	0.201, 0.811

*GAD-7* generalized anxiety disorder-7, *PHQ-9* Patient Health Questionnaire depression-9, *TAS-20* 20-item Toronto Alexithymia Scale, *PDQ-D-20* 20-item Perceived Deficits Questionnaire-Depression, *CI* confidence interval

Bold values indicated statistical significance

We also conducted additional multiple regression analyses considering TAS-20 subscales (DIF, DDF, and EOT) along with the same covariates in relation to subjective cognitive impairment. When repeating the multiple linear regression analyses with TAS-20 subscore as independent variable, sex*PHQ-9 (*β* = 0.630, *t* = 4.688, *p* < 0.001, 95% CI 0.364–0.896) and DDF (*β* = 1.750, *t* = 3907, *p* < 0.001, 95% CI 0.863–2.637) were significantly associated with PDQ-D-20 score.

### Sex difference in association of PDQ-D-20 with clinical variables

As shown in Table 3, after controlling for smoking, age, the PDQ-D-20 score was found to be related to the following characteristics in male patients: DIF (*r* = 0.321, df = 36, *p* = 0.049, *P*_*Bonferroni*_ = 0.294), DDF (*r* = 0.404, df = 36, *p* = 0.012, *P*_*Bonferroni*_ = 0.072), TAS-20 (*r* = 0.391, df = 36, *p* = 0.015, *P*_*Bonferroni*_ = 0.09); however, no variables passed Bonferroni correction (*p* > 0.05). In addition, multiple regression analysis with the stepwise procedure indicated that subjective cognitive impairment was significantly associated with TAS-20 (*β* = 0.705, *t* = 2.575, *p* = 0.014, adjusted *R*^*2*^ = 0.126) (Table 4). When repeating the linear regression analyses with the TAS-20 subscale as an independent variable, DDF (*β* = 2.137, *t* = 2.822, *p* = 0.008, adjusted *R*^*2*^ = 0.151) was significantly associated with PDQ-D-20 score in the male group. In the female group, subjective cognitive impairment was related to the following characteristics: PHQ-9 score (*r* = 0.385, df = 82, *p* < 0.001, *P*_*Bonferroni*_ < 0.01), DIF (*r* = 0.332, df = 82, *p* = 0.002, *P*_*Bonferroni*_ = 0.012), DDF (*r* = 0.379, df = 82, *p* < 0.001, *P*_*Bonferroni*_ < 0.01) and TAS-20 (*r* = 0.3337, df = 82, *p* = 0.002, *P*_*Bonferroni*_ = 0.012), all the characteristics persisted after Bonferroni correction (all *p* < 0.05, Table 3). Furthermore, multiple regression analysis with the stepwise procedure indicated that subjective cognition was significantly associated with PHQ-9 score (*β* = 1.301, *t* = 3.792, *p* < 0.001) and TAS-20 (*β* = 0.436, *t* = 2.473, *p* = 0.015), with adjusted *R*^*2*^ = 0.218 (Table 4). Also, the TAS-20 subscale was used as an independent variable, showing that the PHQ-9 score (*β* = 1.296, *t* = 3.842, *p* < 0.001) and DDF (*β* = 1.624, *t* = 2.884, *p* = 0.005) were significantly associated with PDQ-D-20 score in the female group, with adjusted *R*^*2*^ = 0.237 (Additional file 1: Table S1).

**Table 3:** Correlations between PDQ-D-20 total score and clinical variables in male and female patients with SD

Variables	Male (*n* = 40)		Female (*n* = 86)	
	*r*	*p*	*r*	*p*
GAD-7 score	0.165	0.323	0.080	0.468
PHQ-9 score	0.203	0.222	0.385	< 0.001*
DIF	0.321	**0.049**	0.332	0.002*
DDF	0.404	**0.012**	0.379	< 0.001*
EOT	0.056	0.740	− 0.008	0.944
TAS-20 score	0.391	**0.015**	0.337	0.002*

*GAD-7* generalized anxiety disorder-7, *PHQ-9* Patient Health Questionnaire depression-9, *TAS-20* 20-item Toronto Alexithymia Scale, *PDQ-D* Perceived Deficits Questionnaire-Depression, *DIF* Difficulty Identifying Feelings, *DDF* Difficulty Describing Feeling, *EOT* Externally Oriented Thinking

Bold values indicated statistical significance

*After Bonferroni correction, statistical significance persisted

**Table 4:** Clinical variables independently associated with PDQ-D-20 total score in male and female patients with SD

Model	Independent variable	*β*	SE	*β’*	*t*	*p*	*β* 95% CI	Adjusted *R*^*2*^
Male (*N* = 40)								
1	(Constant)	− 15.424	16.740		− 0.921	0.363	− 49.313, 18.464	0.126
	TAS-20 score	0.705	0.274	0.385	2.575	0.014	0.151, 1.260	
Female (*N* = 86)								
1	(Constant)	19.401	3.749		5.175	< 0.001	11.945, 26.856	0.170
	PHQ-9score	1.483	0.345	0.424	4.296	< 0.001	0.796, 2.169	
2	(Constant)	− 6.178	10.966		− 0.563	0.575	− 27.989, 15.633	0.218
	PHQ-9 score	1.301	0.343	0.372	3.792	< 0.001	0.619, 1.983	
	TAS-20 score	0.436	0.176	0.243	2.473	0.015	0.085, 0.787	

*PHQ-9* Patient Health Questionnaire depression-9, *PDQ-D-20* 20-item Perceived Deficits Questionnaire-Depression, *TAS-20* 20-item Toronto Alexithymia Scale, *SE* standard error, *CI* confidence interval

## Discussion

To the best of our knowledge, this is the first study that explored the sex differences in subjective cognitive impairment and its clinical correlates in Chinese patients with SD. The key findings of our research are: (a) there were no significant differences in the mean TAS-20 score, DIF, DDF, and EOT subconstructs between male and female groups; (b) female patients with SD had significantly higher PDQ-D-20 total score compared to male patients; (c) TAS-20 and DDF subdomain contributed to cognitive impairment in males, whereas both PHQ-9 total score and TAS-20 or DDF subdomain contributed to cognitive impairment in females.

While sex differences in the TAS-20 total score and the three subdomains have been extensively studied, the results remain inconsistent. We found no significant difference in overall and 3 subdomain scores between the two groups. For both student participants (*n* = 870) and psychological patients (*n* = 179) in the Chinese sample, sex differences on the overall TAS-20 score and the three subdomains score were not statistically significant [21]. However, other studies reported that men had significantly higher TAS-20 total scores than women in student or community samples [22–24]. Neumann et al. [25] reported that the TAS-20 total score, DDF, and EOT score were significantly higher in men than women participants. However, the DIF score did not differ between the two sex subgroups. Moreover, they found that age and education contributed to alexithymia [23]. Zhu et al*.* [24] surveyed medical students for alexithymia and found that male participants (*n* = 368) scored higher on the mean TAS-20 total score, DIF, DDF, and EOT compared with female participants (*n* = 1518). Another study reported that girls scored higher than boys on DIF subscales, and the TAS-20 total score did not differ between the two groups in Chinese adolescents [26], which might be due to some factors, such as sample size, different educational backgrounds, cultural differences, age or sex ratio disparity may explain the discrepant results. Therefore, further longitudinal studies with a larger sample are warranted to explore the sex differences of alexithymia in patients with SD.

Interestingly, the mean PDQ-D-20 total score of patients with SD in the present study was similar to the previous study [27], revealing the PDQ-D-20 total score of patients with MDD (30.3 ± 17.91), which was much higher than in community volunteers (9.28 ± 9.63). This finding indicated that patients with SD exhibited cognitive impairment. Furthermore, we found that three factors, i.e., sex, PHQ-9, and TAS-20, were independently associated with subjective cognitive impairment in patients with SD. Interaction between sex and PHQ-9 also contributed to subjective cognitive impairments. Our results showed sex differences in subjective cognitive impairment. Compared with male patients, females had more serious subjective cognitive impairment. It has been demonstrated that the relationship between depression and cognitive impairments varied by gender [28, 29]. Roh et al. reported that middle-aged and older women were more likely to display subjective cognitive impairment than men in the same age groups (OR = 1.59, 95% CI 1.46–1.73) [28]. In addition, Brow*n *et al. [29] reported that the association between depression and subjective cognitive decline-related outcomes was regulated by age and gender. A recent study showed that subjective cognitive decline was more common in women than in men, and the score of cognitive complaints in the women group was significantly higher than in the men group [30]. The symptoms of subjective cognitive decline were assessed by subjective cognitive decline questionnaire-9 (SCD-Q9), where higher scores indicate more symptoms. Lin and his team [31] reported that the female factor contributes to the high SCD-Q9 score. However, another study reported conflicting results, detecting no significant gender differences in cognitive complaint [19]. Furthermore, no significant gender differences were found in the mean PDQ-D-20 total score across various levels of depression among full-time employees [32]. These discrepancies may be due to different diseases, sample representation, or research methods.

More importantly, there were sex differences in the relationship between subjective cognitive impairment and clinical variables of patients with SD. For males, cognitive impairment was significantly associated with the TAS-20 or DIF and DDF subdomain, while for females, cognitive impairment was significantly associated with the TAS-20 total score or the DIF and DDF subdomain, PHQ-9 score. Correlation analysis showed that the male group did not pass the Bonferroni correction. Yet, Bonferroni correction is a very conservative approach, which can easily incorrectly accept the null hypothesis. Notably, we first found that TAS-20 or DDF contributed to cognitive dysfunction in female and male patients with SD. Prior studies showed that alexithymia was correlated with cognitive dysfunction [33]. Galderisi et al. [33] reported that patients with panic disorder had a higher prevalence of alexithymia, lower verbal cognitive abilities, and difficulty inhibiting interference from nonverbal stimuli than the control group. In their study, Santorelli et al. reported that greater alexithymia and DDF were associated with poorer verbal executive function in older subjects (aged 61–92) but not in younger adults (aged 18–30) [20]. Correro et al. assessed executive functioning using neuropsychological testing and reported that alexithymia was significantly associated with age-related cognitive decline. Moreover, they found that high EOT contributed to poorer memory performance and DIF contributed to poor executive function in younger and older healthy adults [34].

Consistent with some previous studies [27, 35–37], we found that subjective cognitive impairment was significantly associated with the severity of depressive symptoms in the whole sample. In addition, Manit et al. [36] reported that PHQ-9 and PDQ-D were moderate to highly correlated (*r* = 0.69). However, one study reports did not find an association between cognitive impairment and the severity of depressive symptoms [38]. Nonetheless, this research did not explore sex differences in the relationship between subjective cognitive impairments and psychopathological symptoms. Interestingly, we found that the PHQ-9 score was associated with cognitive dysfunction in female patients but not in male patients, which was inconsistent with previous reports showing that the mean PDQ-D-20 score in men did not significantly differ from women across various levels of depression [32]. These discrepant results may be due to sampling representation, different stages of disease (acute episode vs. remission period), duration of illness, and exposure to antidepressant medication. For example, Galimberti et al. reported that a longer duration of untreated illness was associated with worse cognitive function during depression [39, 40].

The mechanisms of sex differences in the relationship between cognitive dysfunction and psychopathological symptoms remain unclear. Some factors, including social and psychological factors and biological factors, may contribute to sex differences. For example, gender differences in sex hormones [41] may contribute to varying degrees of cognitive impairment. In addition, the absence of the duration of the illness also contributed to bias. Unfortunately, as we did not examine the level of sex hormones or include this factor (duration of illness) in our study, further investigation is warranted to clarify the mechanism.

### Limitations

There are several limitations in the present study. First, an important limitation is the absence of a healthy control group; hence, the reported findings should be interpreted with caution. Second, it is well known that cognitive symptoms are associated with some clinical variables, such as duration of illness, acute episode, or remission period. Unfortunately, we did not examine these factors. Therefore, further studies with large sample sizes and these relevant factors should be conducted to confirm these findings. Third, as it is difficult to guarantee the quality of the online survey, we only used a patient-reported questionnaire in this study, which could affect the accuracy of the data. Forth, due to the nature of the cross-sectional study design, causality between cognitive impairment and the associated risk factors cannot be inferred, which should be further investigated by future longitudinal studies.

### Perspective and significance

The present study found sex differences in subjective cognitive impairment and a different association between subjective cognitive impairment and clinical correlates in females and males. Female patients had a higher PDQ-D-20 score than male patients, indicating worse cognitive impairment in females. Interestingly, subjective cognitive impairment was correlated with the TAS-20 and PHQ-9 scores in female patients, while only the TAS-20 score was in male patients. Therefore, improving the severity of depressive symptoms ameliorated cognitive dysfunction in females. Further studies should consider the sex role when assessing cognitive symptoms since its association with the clinical symptoms was distinct between males and females. In addition, further follow-up and controlled prospective studies with a large sample size could help clarify the interrelationship between cognitive impairment and clinical symptoms.

## Supplementary Information

**Additional file 1: Table S1.** Clinical variables independently associated with PDQ-D-20 score in male and female patients with SD.

## Data Availability

The datasets used and/or analyzed in the current study are available from the corresponding author upon reasonable request.
