# Supplementary material for: Sex differences in subjective cognitive impairment and clinical correlates in Chinese patients with subthreshold depression
Source: Biol Sex Differ. 2023 Feb 13;14:6. doi: 10.1186/s13293-023-00488-w (PMC9926784; doi:10.1186/s13293-023-00488-w)
Supplement: Supplementary file 1 — Additional file 1: Table S1. Clinical variables independently associated with PDQ-D-20 score in male and female patients with SD. [file 13293_2023_488_MOESM1_ESM.doc]

Table S1 Clinical variables independently associated with PDQ-D-20 score in male and female patients with SD

| Male（N=40） | | | | | | | | |  |
| --- | --- | --- | --- | --- | --- | --- | --- | --- | --- |
| model | Independent variable | β | | SE | β’ | t | P | β95%CI | adjusted R^2^ |
| 1 | (constant) | -3.580 | | 11.149 |  | -.321 | 0.750 | -26.150,18.989 | 0.151 |
|  | DDF | 2.137 | | 0.757 | 0.416 | 2.822 | 0.008 | 0.604,3.670 |  |
| Female（N=86） | | | | | | | | |  |
| model | Independent variable | | β | SE | β’ | t | P | β 95%CI | adjusted R^2^ |
| 1 | (constant) | | 19.401 | 3.749 |  | 5.175 | < 0.001 | 11.945,26.856 | 0.170 |
|  | PHQ-9 score | | 1.483 | 0.345 | 0.424 | 4.296 | < 0.001 | 0.796,2.169 |  |
| 2 | (constant) | | -3.097 | 8.591 |  | -0.360 | 0.719 | -20.183,13.990 | 0.237 |
|  | PHQ-9 score | | 1.296 | 0.337 | 0.371 | 3.842 | < 0.001 | 0.625,1.967 |  |
|  | DDF | | 1.624 | 0.563 | 0.278 | 2.884 | 0.005 | 0.504,2.744 |  |

**Abbreviations:**PHQ-9=Patient Health Questionnaire depression-9,PDQ-D-20=20-item Perceived Deficits Questionnaire-Depression,DDF=Difficulty Describing Feelings,SE=Standard error,CI=Confidence Interval.
